# QSAR, molecular docking, design, and pharmacokinetic analysis of 2-(4-fluorophenyl) imidazol-5-ones as anti-breast cancer drug compounds against MCF-7 cell line

**DOI:** 10.1007/s10863-020-09858-0

**Published:** 2020-11-27

**Authors:** Hadiza Abdulrahman Lawal, Adamu Uzairu, Sani Uba

**Affiliations:** Department of Chemistry, Ahmadu Bello University, P.M.B, Zaria, 1044 Nigeria

**Keywords:** QSAR analysis, 2-(4-fluorophenyl) imidazol-5-ones, Ligand-based design, Pharmacokinetics, Breast cancer

## Abstract

The anti-proliferative activities of Novel series of 2-(4-fluorophenyl) imidazol-5-ones against MCF-7 breast cancer cell line were explored via in-slico studies which includes Quantitative structure–activity relationship QSAR, molecular docking studies, designing new compounds, and analyzing the pharmacokinetics properties of the designed compounds. From the QSAR analysis, model number one emerged the best as seen from the arithmetic assessments of (R^2^) = 0.6981, (R^2^_adj_) = 0.6433, (Q^2^) = 0.5460 and (R^2^_pred_) of 0.5357. Model number one was used in designing new derivative compounds, with higher effectiveness against estrogen positive breast cancer (MCF-7 cell line). The Molecular docking studies between the derivatives and Polo-like kinases (Plk1) receptor proved that the derivatives of 2-(4-fluorophenyl) imidazol-5-ones bind tightly to the receptor, thou ligand 24 and 27 had the highest binding affinities of −8.8 and − 9.1 kcal/mol, which was found to be higher than Doxorubicin with a docking score of −8.0 kcal/mol. These new derivatives of 2-(4-fluorophenyl) imidazol-5-ones shall be excellent inhibitors against (plk1). The pharmacokinetics analysis performed on the new structures revealed that all the structures passed the test and also the Lipinski rule of five, and they could further proceed to pre-clinical tests. They both revealed a revolution in medicine for developing novel anti-breast cancer drugs against MCF-7 cell line.

## Introduction

Cancer is described by uncontrolled cell proliferation, thereby affecting the surrounding tissue, and over again spreading throughout the body. It is a complicated disease. (Bajaj et al. 2018). Despite the vast high-tech and social enhancement, cancer remains the most alarming disease and a leading cause of pain and death in humans (Bhaumik et al. 2019).

Among women, after lung cancer, cancer of the breast is the second source of mortality. About 40,610 women passed away from cancer of the breast and about 252,710 more diagnoses were projected in 2017 (Bajaj et al. 2018). Cancer of the breast accounts for about 24% of all cancer types in females (Xiao et al. 2018). A likely population of people with breast cancer has similar features such as older age, lack of prolonged breast feeding, adding weight, overdue age at first birth, lack of exercise, etc. (Liu et al. 2018). Detailed investigation of pathways and mechanisms on how cancer spreads and discovering many anti-cancer agents have made a breakthrough in the treatment of cancer (Bhaumik et al. 2019).

Luminal type of breast cancer is Estrogen receptor (ER)−/progesterone receptor (PR) - the positive type which is caused by the overexpression of estrogen receptor α (ERα). It is made up of about 70% of the mammary tumor patients tagged as ER-positive (ER+). The endless stimulation of ERα by estrogens induce the multiplication of cancer cells, MCF-7 cell line (Jordan et al. 2007). The master mitotic regulator, Polo-like kinase 1 (Plk1), is an important gene cell division and a known cancer drug target. It is found overexpressed in a large collection of different cancer types and this tumoral overexpression often correlates with poor patient prognosis (De Cárcer 2019).

In the study of (Sanhaji et al. 2012), it showed that Polo-like kinases (Plk1) causes destructive proliferation in tumor cells and strongly stimulates the development of cell circle. Plk1 overexpression allows cells to supersede barriers, causing genomic uncertainty and stimulating the alteration of mammalian cells. Plk1 was proven as amongst the utmost striking receptor for breast cancer treatment. Plk1 mediates estrogen receptor (ER) which regulates gene overexpression in human breast cancer cells. Recently, (Abo-Elanwar et al. 2019) reported novel Thirty-nine derivatives of Imidazolones connected to chalone moiety which showed a great anti-breast cancer activity against MCF-7 cell line. Imidazole and its derivatives have much significance in both natural products and synthetic molecules. The exclusive molecules have great electron-rich features which allow them to bind easily with diverse enzymes and receptors, thus showing wide anti-proliferative activities (Zhang et al. 2020). Several activities such as anticancer, antimicrobial (Premakumari et al. 2014), cardio-activity, and angiotensin II receptor antagonistic activity have been described explicitly in compounds containing imidazolone moiety (Abo-Elanwar et al. 2019).

Chemotherapy remains one of the best and fast clinical options though it is often limited due to undesirable toxic effect including weight loss, fatigue, nausea, loss of appetite, and so on, making it urgent to develop more effective drug candidates with less toxicity to eradicate this disease (Iqbal et al. 2019). The computer-aided drug design approach saves time and ensures better effectiveness of the drug candidate. This research would be aimed at exploring the novel derivatives of imidazolone by building a mathematical model (QSAR) that predicts the anti-proliferative activities from its compound and using plk1 receptor with the derivatives to understand their interactions via molecular docking studies towards anti-breast cancer drug discovery, concentrating on breast cancer treatment with less toxicity and more effectiveness.

## Methodology

### Computer applications

The software includes; Chemdraw version 12.0.2, Spartan’14 (version 1.1.2), Material studio (V8) software, Pyrex software, PADEL V2.20, DTC data lab software version, and Auto dock visualizer version 4.2.

### QSAR studies

#### Dataset

39 derivatives of Imidazolones connected to chalone moiety with anti-proliferative activities (IC_50_) on MCF-7 cancer cell lines were obtained from (Abo-Elanwar et al. 2019) writings. The anti-proliferative activities were measured in inhibitory concentration (IC_50_) and then converted to the logarithm scale (pIC_50_). The tabulated form of the IC_50_ is measured in concentration of micromolar (μM) and the pIC_50_ is shown in Table 1.

$$ {\mathrm{pIC}}_{50}=-\log 10\ \left({\mathrm{IC}}_{50}\ \mathrm{x}\ {10}^{-6}\right). $$

**Table 1:**
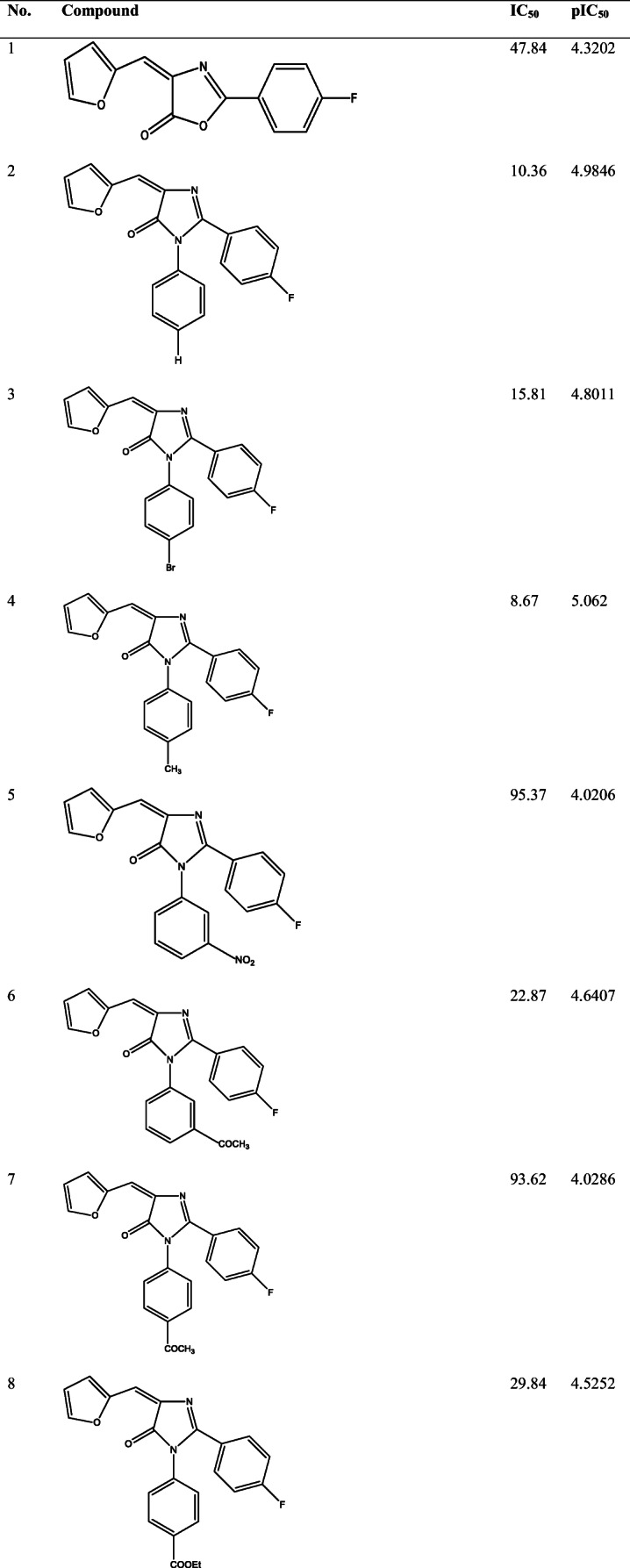

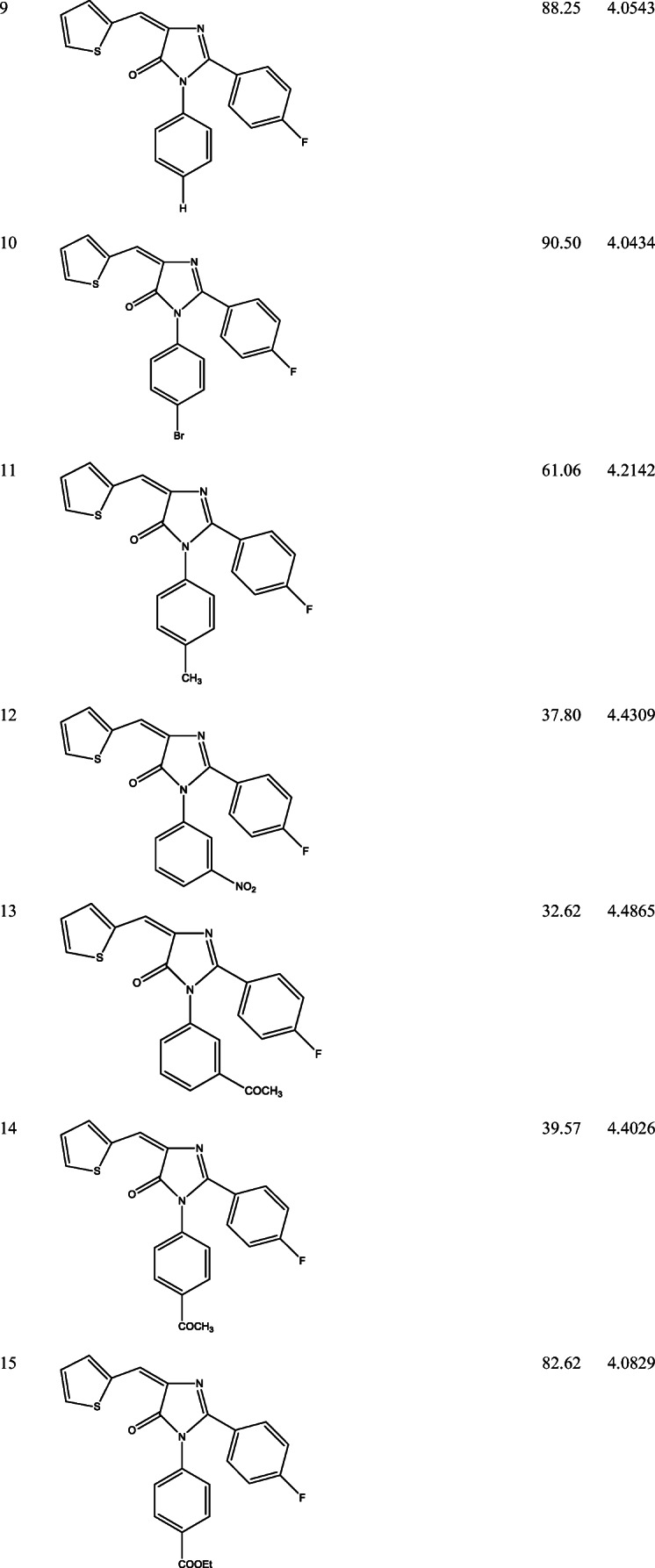

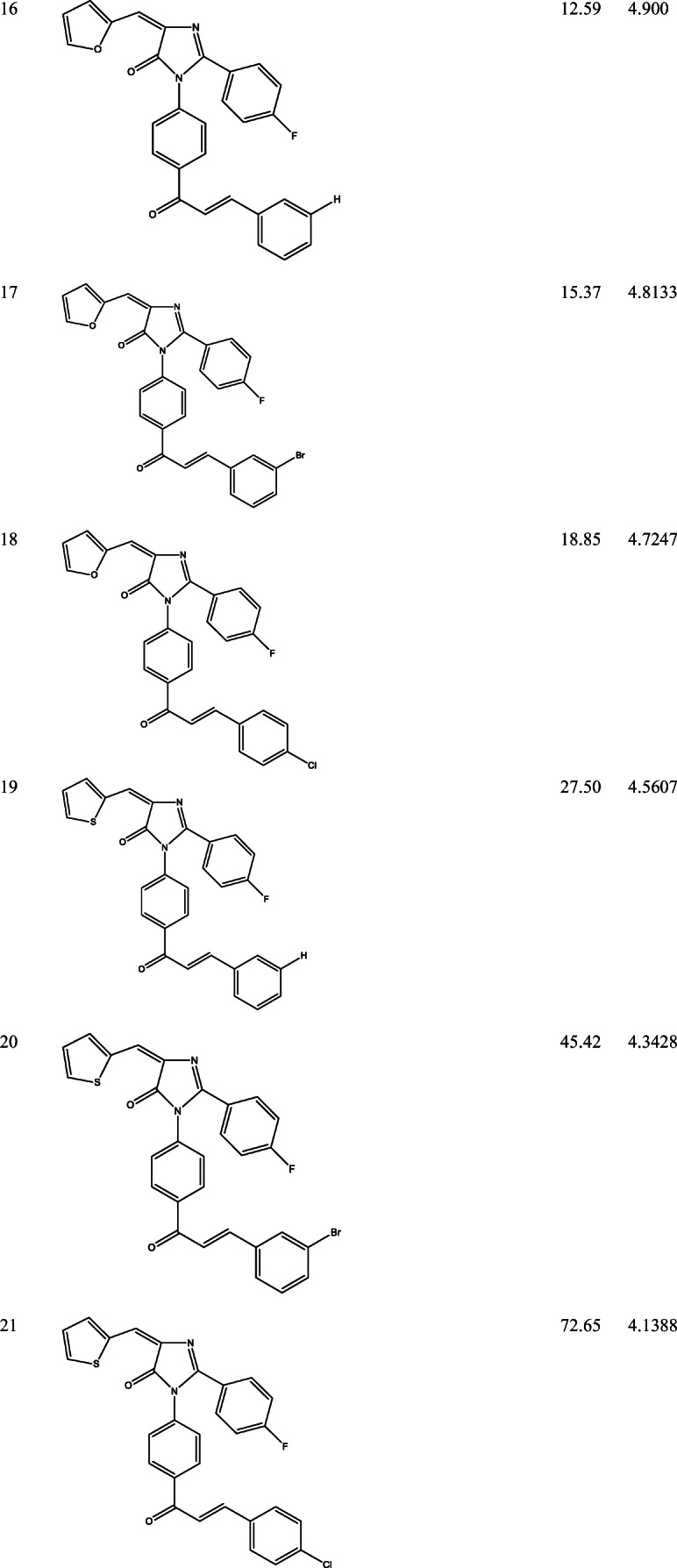

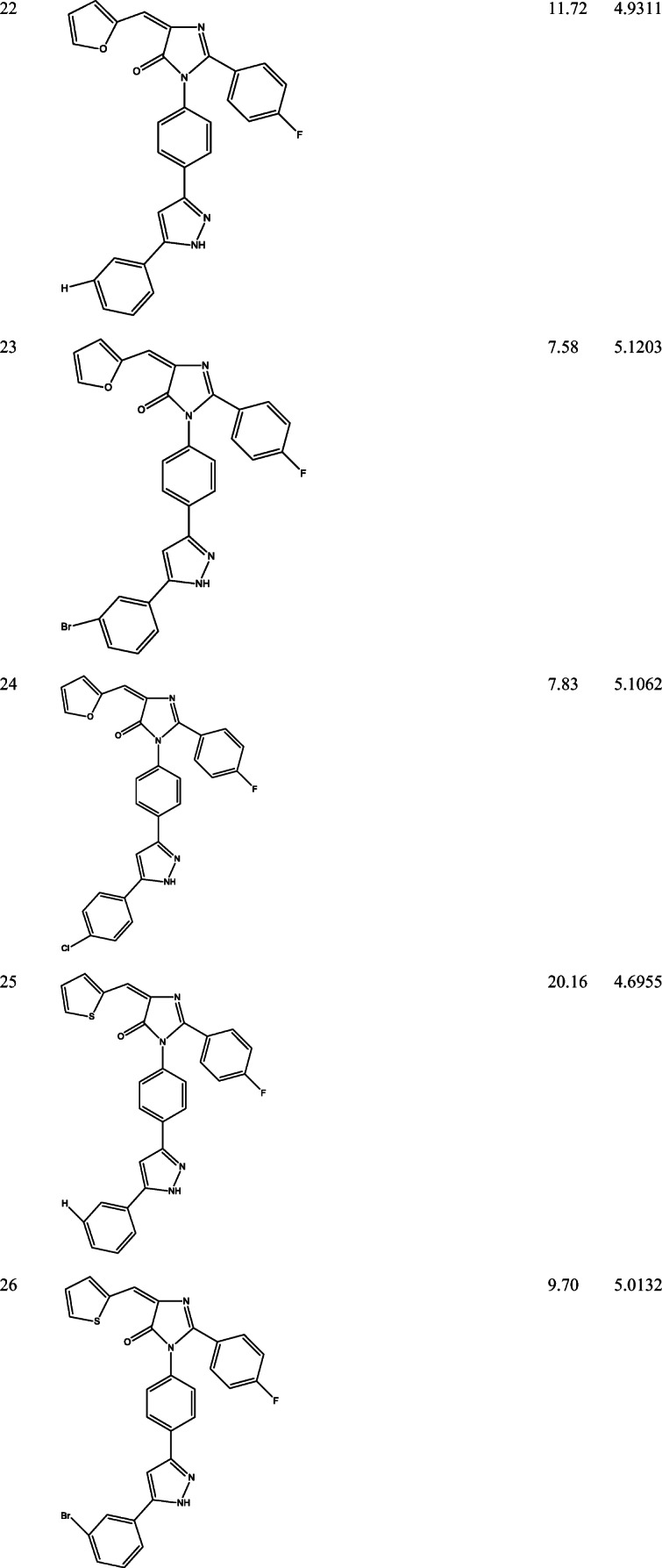

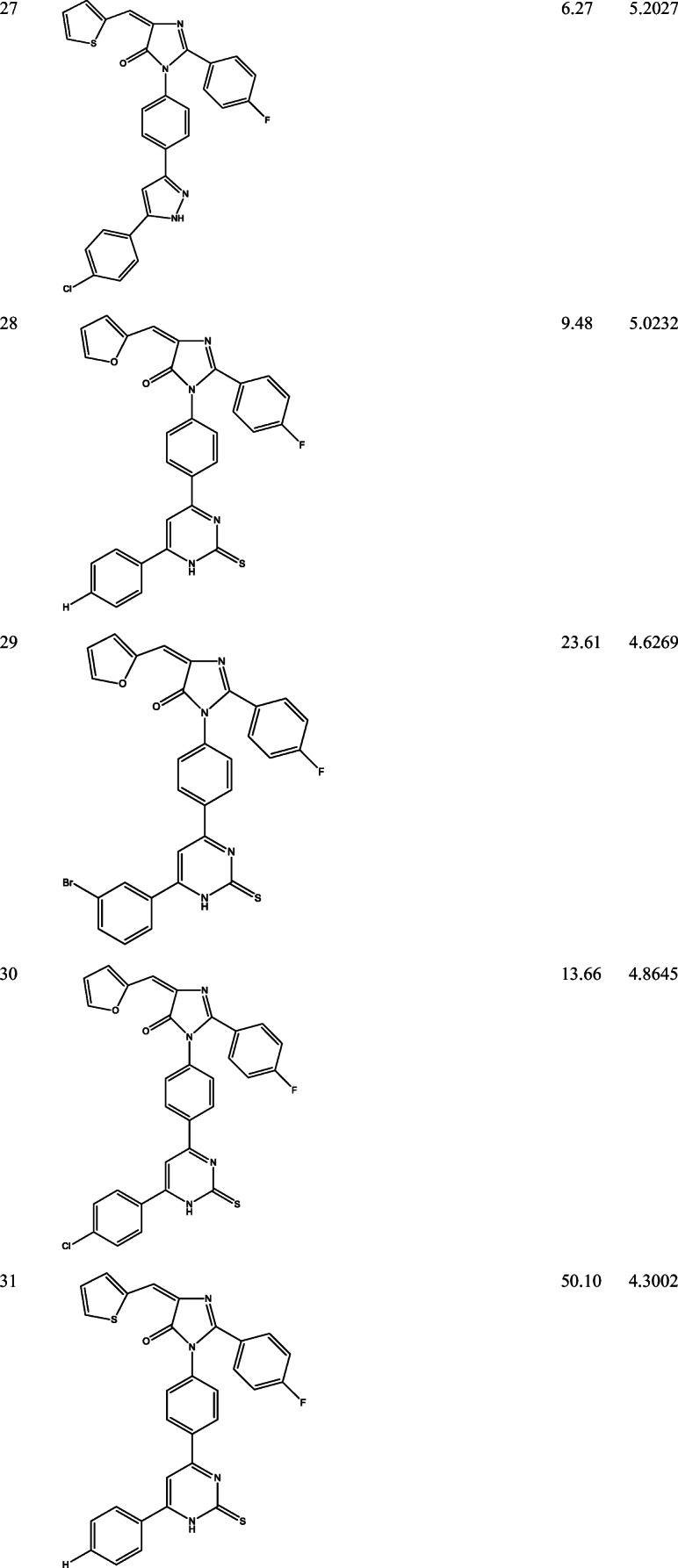

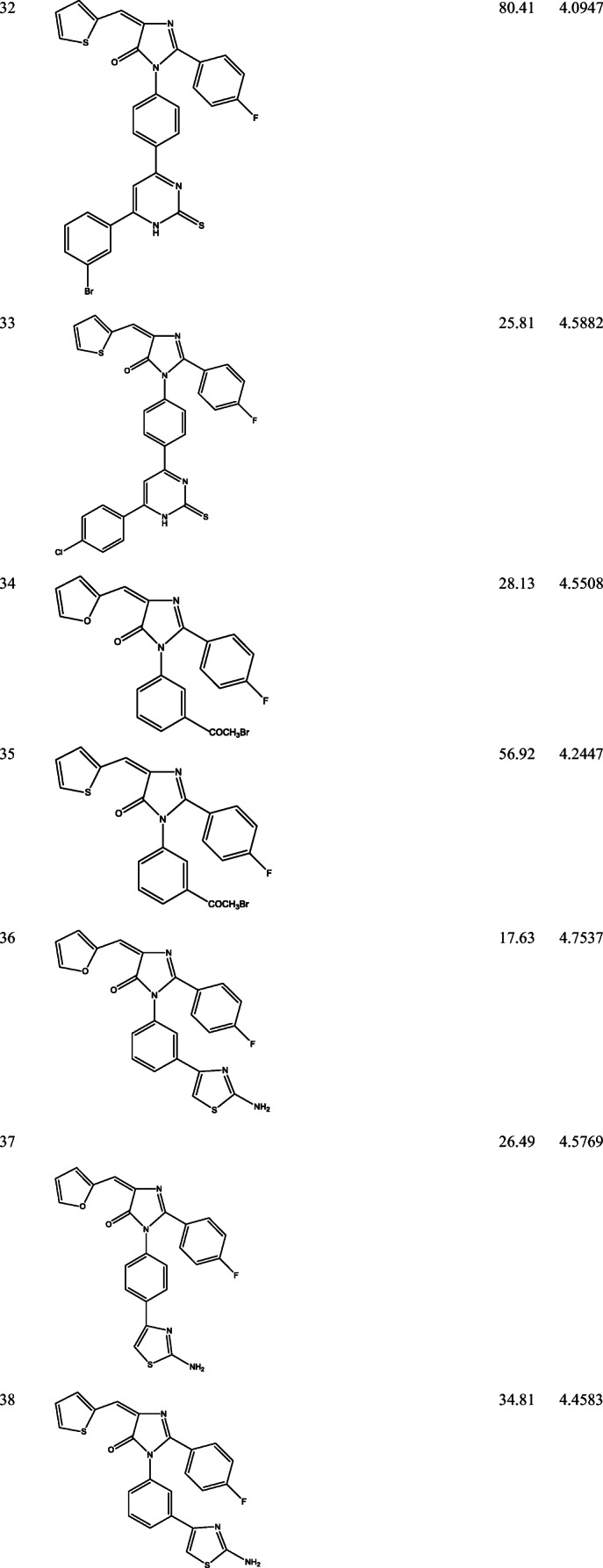

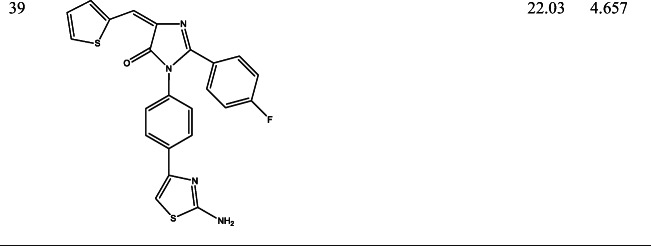
2-(4-fluorophenyl) imidazol-5-ones derivatives and its activities

#### Molecular optimization

The geometric optimization is executed such that the countable electronic and molecular parameters could depict the original physicochemical properties of the observed molecule. (Putri et al. 2019). The derivatives were sketched using Chemdraw (V12.0.2) software in 2D format. They were then converted to 3D format and further optimized using Spartan 14 (V1.1.4) software, with the parameters, Density Functional Theory (DFT) at B3LYP, 6-31G/ basis set (Ibrahim et al. 2018; Abdullahi et al. 2019).

#### Molecular descriptors

Pharmaceutical Data Exploration Laboratory Software V (2.20) was used in calculating molecular descriptors for the thirty-nine (39) optimized compounds of Imidazolones derivatives which were then converted to SDF format after optimization. (Yap 2011).

#### Pretreatment and division of data set

The outcomes obtained from PADEL-software were pretreated to remove persistent values and undesirable descriptors using Data Pre-treatment software GUI 1.2. (Abdulrahman et al. 2020). Kennard-Stone algorithm (Kennard and Stone 1969) method divided the derivatives into 27 calibration and 12 validation sets to get a mathematical equation.

#### QSAR equation

Material studio software (V8) was used in constructing a model with Genetic Function Approximation (GFA) procedure. The dependent variable is the anti-proliferative activities (pIC_50_) and the independent variable is model parameters.

#### Validating the equation (internal)

The validity of the built model should be tested on an external set of data that has not yet been used during the process of developing the model (Tropsha et al. 2003). The built equations were evaluated using the Friedman formula; (Friedman 1991).

$$ \mathrm{LOF}=\kern0.5em \frac{SEE}{M{\left[1-\beta \left(\frac{c+d\times p}{M}\right)\right]}^2} $$

Where: *SEE* is the Standard Estimated Error. If SEE is small, it suggests an enhanced equation. SEE is expressed below;

$$ \mathrm{SEE}=\sqrt{\frac{{\left({Y}_{exp}-{Y}_{pred}\right)}^2}{N-P-1}} $$

*d* equals user-defined smoothing parameter, *C* equals to the sum of the model terms, *M* equals the sum of train set compounds and *p* is the equation parameters (Ibrahim et al. 2018). The correlation coefficient (R^2^) accounts for the fragment of the total difference of the equation. The nearer the R^2^ value is to 1, the more enhanced the model is built. R^2^ is given as:

$$ {\mathrm{R}}^2=1-\left[\frac{\sum {\left({Y}_{exp}-{Y}_{pred}\right)}^2}{\sum {\left({Y}_{exp}-{Y}_{training}\right)}^2}\right] $$

Where *Y*_*exp*_ and *Y*_*pred*_ are the means of biological and calculated activities of the calibration set (Tropsha et al. 2003).

R^2^ rises as the number of descriptors increase. Hence R^2^ does not assure the effectiveness of the equations. R^2^_adj_ is used to reconfirm the strength and effectiveness of the equation.

$$ {\mathrm{R}}_{\mathrm{adj}}^2=\frac{R^2-P\left(n-1\right)}{n-p+1} $$

Where p and n are the descriptors from the equation and calibration set. Validation coefficient test (Q^2^_cv_) was used in assessing the robustness of the model and prediction power of the derivatives, it’s given as:

$$ {\mathrm{Q}}_{\mathrm{cv}}^2=1-\left[\frac{\sum {\left({Y}_{pred}-{Y}_{exp}\right)}^2}{\sum {\left({Y}_{exp}-{Y}_{mint\mathrm{r} aining}\right)}^2}\right] $$

Y_mintraining_ Y_exp_, and Y_pred_ equals to the mean activities (pIC_50_) of calibration set, bio-activities (IC_50_) and calculated of the calibration set (Brandon and Orr 2015).

#### External model validation

##### Mean effect

The mean effect shows the descriptors or model parameters that influence the generated equation.

The symbols on the model parameters show the various impact of each parameter in the overall derived equation, either an increase or decrease of the model parameter. Thus it’s expressed as;

$$ Mean\ effect=\frac{\beta_j{\sum}_i^n{D}_j}{\sum m\left({\beta}_j{\sum}_i^n{D}_j\right)} $$

Where *m* equals the model parameters, *B*_*j*_ equals to descriptors coefficient *j*, *n* equals to the prediction set molecules and *D*_*j*_ is the matrix value of the model parameter in the prediction set (Minovski et al. 2013).

##### Variance inflation factor (VIF)

The VIF takes into account the amount of co-linearity amongst the descriptors in an equation. It is calculated as

$$ VIF=\frac{1}{\left(1-{R}^2\right)} $$

R^2^ is the correlation coefficient. (Myers 1990).

The greater the value, the bigger the link amongst the model parameters. The VIF values of less than 10 show the equation is stable while the values above 10 indicate the equation is not effective and cannot be used.

##### Applicability domain

The applicability domain approach is aimed at estimating independently, the reliability of every generated equation (Eriksson et al. 2003). Model validation should be within the training domain and the compounds need to be assessed as fitting within the domain to ascertain the model. The applicability domain is evaluated by the leverage values for each compound. Leverage defines the applicability domain of the built equation (Veerasamy et al. 2011). It is given as;

$$ {H}_i={x}_i\ {\left({X}^TX\right)}^{-k}\ {x_i}^T\ \left(i={K}^{\dots },P\right) $$

Where *X*^*T*^ is the matrix transpose of *X* used in constructing the equation, *X*_*i*_ is the matrix of calibration set of *I* and *X* is the *n x k* matrix of train set descriptors. (d*) is the warning leverage, d* searches for outliers. It is shown as;

$$ \mathrm{d}\ast =\frac{3\left(k+1\right)}{n} $$

*k* equals to the total model parameters and *n* equals to the calibration compounds. William’s plot (A plot of standardized values vs. leverages) of the calibration and validation compounds. Molecules found in the warning leverages within the graph are the calculated molecules.

#### Quality assurance model generated

Table 2 shows the least required values in assessing the mathematical equation (Ibrahim et al. 2018). The table parameters were used in conforming to the effectiveness and prediction power of the derived equations.

**Table 2:** Recommended values for evaluating QSAR equations

Character	Name	Value
R^2^	Coefficient of determination	≥0.6
P_(95%)_	Confidence interval at 95% confidence level	<0.05
Q^2^	Squared cross-validation coefficient	≥0.5
R^2^-Q^2^	Difference between R^2^ and Q^2^	<0.3
N_test set_	Least number of the test set	≥5
R^2^_ext_	Coefficient of determination of external validation	≥0.5

### Molecular docking studies

Five compounds with high pIC_50_ underwent molecular docking studies with the receptor Polo-like kinase 1(PKL1) in complex with B16727. The receptor used was obtained from Protein Data Bank (Code: 3FC2) and was set using Discovery studio software, the ligand (compounds) were also converted to PBD format as shown in Fig. 4. Autodock Vina of Pyrx software was employed in calculating the binding affinity of the ligand and receptor (Abdulfatai et al. 2018).

### Pharmacokinetics (drug-likeness)

SwissADME; an online tool, used in investigating the ADME property physicochemical, pharmacokinetic, and medicinal chemistry responsiveness of smalls compounds (Daina et al. 2017) was employed in assessing the Pharmacokinetic parameters of the new structures.

Again, the designed compounds were checked for their adaptability with Lipinski’s rule of five (Hou et al. 2019), well-used criteria to comprehend if a compound can be taken orally or not, such as molecular weight (MW) ≤ 500, octanol/water partition coefficient (AlogP) ≤ 5, number of hydrogen bond donors (HBDs) ≤ 5 and number of hydrogen bond acceptors (HBAs) ≤ 10.6. According to the rule of five, a compound cannot be taken orally if it does not meet up to two or rules out of the rules of five (Guan et al. 2019).

## Results and discussion

### QSAR of 2-(4-fluorophenyl) imidazol-5-ones derivatives

QSAR examination was used to verify the relationship of 2-(4-fluorophenyl) imidazol-5-ones derivatives with its anti-proliferative activities. Using the Genetic Function Approximation (GFA) method, four QSAR equations were built to predict the anti-proliferative activities of imidazole derivatives. From both internal and external validation parameters, model number 1 passed with correlation coefficient squared (R^2^) of 0.6981, correlation coefficient adjusted squared (R^2^_adj_) of 0.6433, cross-validation coefficient (Q^2^) of 0.5460 and external validation (R^2^_pred_) of 0.5390. Tables 3 and 4 shows how the external validation of model 1 was calculated using the validation set (test set) and model descriptors.

- Model 1

**Table 3:** External validation of equation 1

Name	pIC_50_	MATS4e	GATS5e	SpMax4_Bhs	RDF150u	Y_pred_	Y_pred_-Y_obs_
23	5.1203	−0.1068	0.8685	3.9563	5.5912	4.9935	0.1268
24	5.1062	−0.1214	0.8301	3.9563	4.7605	4.9494	0.1568
27	5.2027	−0.1666	0.9511	3.8468	6.6068	4.7426	0.4601
33	4.5882	−0.1687	0.8792	3.8317	0.8057	4.4399	0.1483
35	4.2447	−0.1646	0.9304	3.8245	0.0040	4.2702	−0.0255
36	4.7537	−0.1093	0.8139	3.9606	1.24E-28	4.6337	0.1199
37	4.5769	−0.1197	0.8164	3.9607	0.3669	4.6078	−0.0309
38	4.4583	−0.1590	0.9474	3.7874	1.90E-17	4.3050	0.1533
39	4.6570	−0.1725	0.9504	3.7880	1.6951	4.3798	0.2772
6	4.6407	−0.1135	0.7806	3.9553	4.89E-47	4.7065	−0.0658
7	4.0286	−0.1231	0.8095	3.9553	2.53E-33	4.5857	−0.5571
8	4.5252	−0.1680	0.8066	3.9661	0.9960	4.4381	0.0871

**Table 4:** Calculation on external validation of equation 1 (continued)

(Y_pred_-Y_obs_)^2^	Y_mintrn_	(Y_mintrn_-Y_obs_)	(Y_mintrn_-Y_obs_)^2^
0.0161	4.5364	0.5839	0.3409
0.0246	4.5364	0.5698	0.3247
0.2117	4.5364	0.6663	0.4430
0.0210	4.5364	0.0518	0.0027
0.0007	4.5364	−0.2917	0.0851
0.0144	4.5364	0.2173	0.0472
0.0009	4.5364	0.0405	0.0016
0.0235	4.5364	−0.0781	0.0061
0.0769	4.5364	0.1206	0.0145
0.0043	4.5364	0.1043	0.0109
0.3104	4.5364	−0.5078	0.2579
0.0076	4.5364	−0.0112	0.0001

∑(Y_ob_ − Y_pred_)^2^ = 0.713008 ∑(Y_obs_ − Y ®_train_)^2^ = 1.535707∴ R^2^*test* = 1 − (0.7130/ 1.5357) = 0.5357

$$ {\mathrm{pIC}}_{50}=4.888518176\ast \mathrm{MATS}4\mathrm{e}-2.570261057\ast \mathrm{GATS}5\mathrm{e}-1.514002889\ast \mathrm{SpMax}4\_\mathrm{Bhs}+0.086137333\ast \mathrm{RDF}150\mathrm{u}+13.256220911 $$

- Model 2

$$ {\mathrm{pIC}}_{50}=-0.018183153\ast \mathrm{ALogp}2+4.669978294\ast \mathrm{MATS}4\mathrm{e}-1.245497827\ast \mathrm{GATS}5\mathrm{e}+0.065416696\ast \mathrm{RDF}150\mathrm{u}+6.326485838 $$

- Model 3

$$ {\mathrm{pIC}}_{50}=-0.021417358\ast \mathrm{ALogp}2+2.673511925\ast \mathrm{MATS}4\mathrm{e}-1.523695399\ast \mathrm{GATS}4\mathrm{e}+0.061648777\ast \mathrm{RDF}150\mathrm{u}+6.593640317 $$

- Model 4

$$ {\mathrm{pIC}}_{50}=4.242442241\ast \mathrm{MATS}4\mathrm{e}-1.572590496\ast \mathrm{GATS}5\mathrm{e}+0.065174802\ast \mathrm{RDF}150\mathrm{u}-0.392069439\ast \mathrm{P}1\mathrm{m}+6.739118905 $$

The biological, calculated and the residual values of 2-(4-fluorophenyl) imidazol-5-ones compounds are seen in Table 5.

**Table 5:** The bio-activities (pIC_50_), prediction inhibition and residual of model 1

Name	pIC_50_	Predicted IC_50_	Residual
1	4.3202	4.3219	−0.0017
2	4.9846	4.7403	0.2443
3	4.8011	4.6737	0.1274
4	5.0262	5.0411	0.0209
5	4.0206	4.2734	−0.2529
6*	4.6407	4.7065	−0.0658
7*	4.0286	4.5857	−0.5571
8*	4.5252	4.4381	0.0871
9	4.0543	4.1766	−0.1223
10	4.0434	4.1436	−0.1002
11	4.2142	4.5352	−0.3210
12	4.4309	4.0666	0.3643
13	4.4865	4.4213	0.0652
14	4.4026	4.2774	0.1252
15	4.0829	4.1101	−0.0272
16	4.9000	4.8921	0.0079
17	4.8133	4.8557	−0.0424
18	4.7247	4.9270	−0.2023
19	4.5607	4.3625	0.1982
20	4.3428	4.1947	0.1481
21	4.1388	4.4897	−0.3509
22	4.9311	4.9916	−0.0605
23*	5.1203	4.9935	0.1268
24*	5.1062	4.9494	0.1568
25	4.6955	4.7353	−0.0398
26	5.0132	4.6610	0.3522
27*	5.2027	4.7426	0.4601
28	5.0232	4.7887	0.2345
29	4.6269	4.7330	−0.1061
30	4.8645	4.7518	0.1127
31	4.3002	4.3924	−0.0922
32	4.0947	4.3760	−0.2813
33*	4.5882	4.4399	0.1483
34	4.5508	4.5509	−0.0005
35*	4.2447	4.2702	−0.0255
36*	4.7537	4.6338	0.1199
37*	4.5769	4.6078	−0.0309
38*	4.4583	4.3050	0.1533
39	4.6570	4.3798	0.2773

The compounds with (*) are the validation compounds while the compounds without (*) are the calibration set

The descriptors obtained from the mathematical model 1 were defined and classified as seen in Table 6.

**Table 6:** Definition of descriptors and their classes for model 1

Name	Definition	Class
MATS4e	Moran autocorrelation - lag 4 / weighted by Sanderson electronegativities.	2D
GATS5e	Geary autocorrelation - lag 5 / weighted by Sanderson electronegativities.	2D
SPMAX4_Bhs	The largest absolute eigenvalue of Burden modified matrix - n 4 / weighted by relative I-state.	2D
RDF150U	Radial distribution function-150 / unweighted.	2D

Further statistical analysis was carried out on the model parameters to find the correlation between the individual descriptors and also the impact of each descriptor in the model. The results are shown in Table 7.

**Table 7:** Statistical analysis of model 1 parameters

	MATS4e	GATS5e	SpMax4_Bhs	RDF150u	VIF	Mean Effect
MATS4e	1	−0.36547	0.511519	0.208789	1.38903	0.07868
GATS5e	−0.36547	1	−0.60954	0.310998	3.140572	0.268682
SpMax4_Bhs	0.511519	−0.60954	1	0.338777	3.267216	0.669708
RDF150u	0.208789	0.310998	0.338777	1	2.220033	−0.01707

**Fig. 1:**
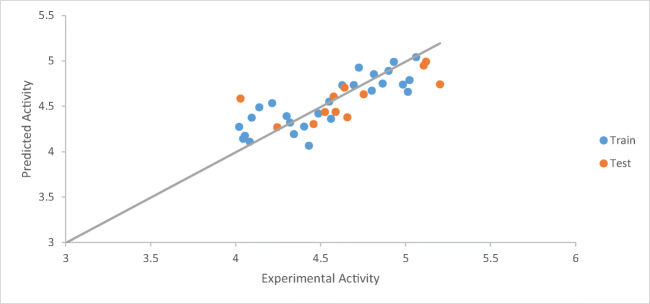
Plot of calculated activity versus inhibition concentration (biological activities)

A graph of the calculated activities against the biological activities was drawn to show the relationship between derivatives as seen in Fig. 1.

**Fig. 2:**
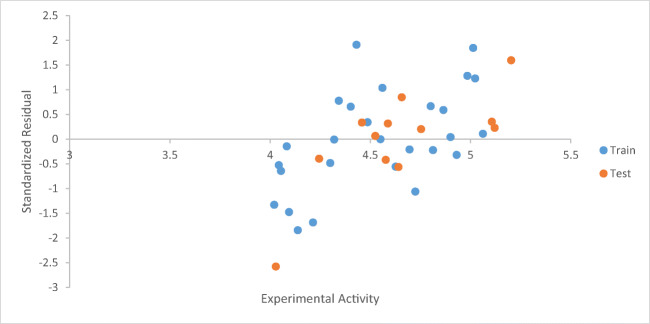
Plot of standardized residual against bioactivities (Experimental activity).

Figure 2 shows a plot of standardized residual values versus the experimental activities of the derivatives.

**Fig. 3:**
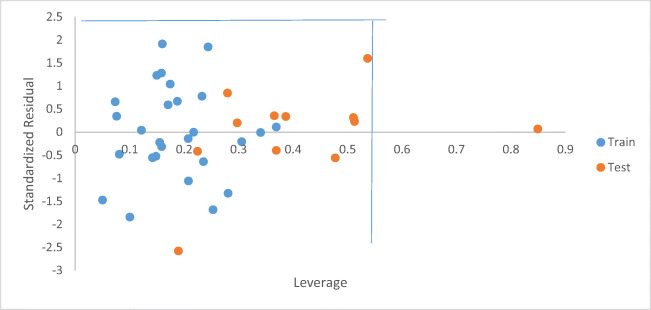
The William’s plot

**Fig. 4:**
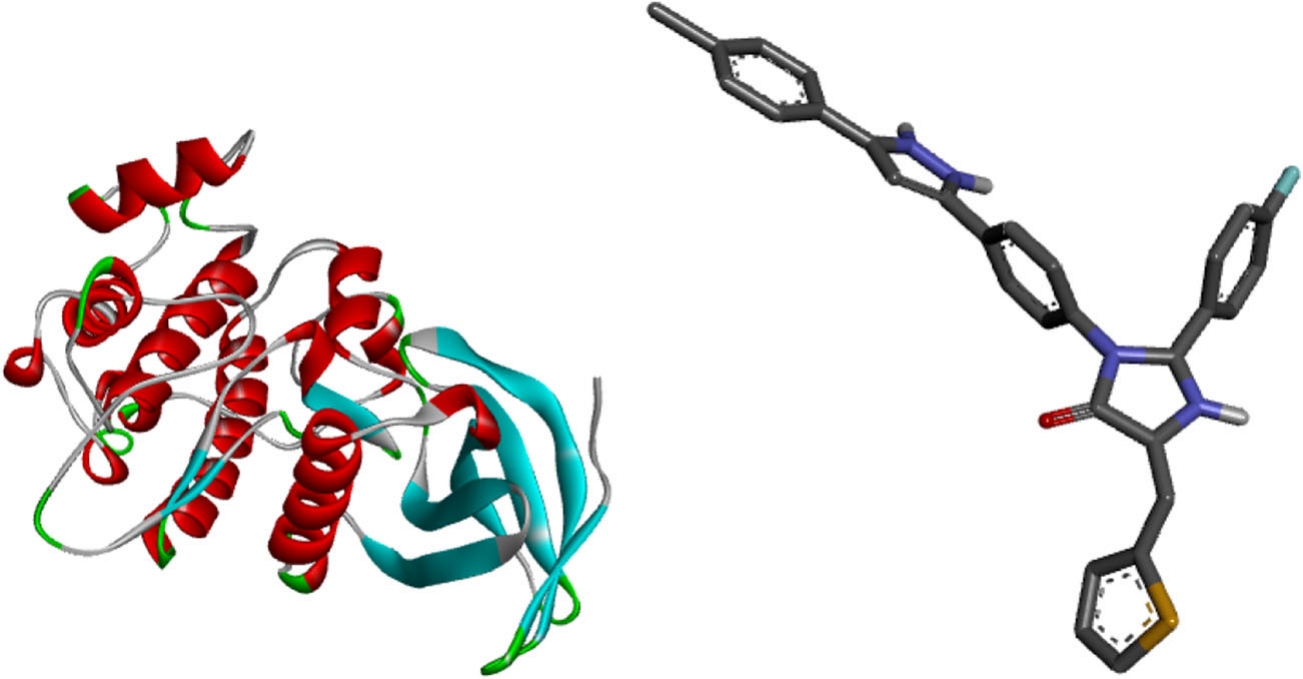
3D representation of prepared ligand and receptor

A graph of standardized residual against the leverages was plotted to show the derivatives that fell within the applicability domain as seen in Figs. 3 and 4.

### Molecular docking analysis

**Fig. 5:**
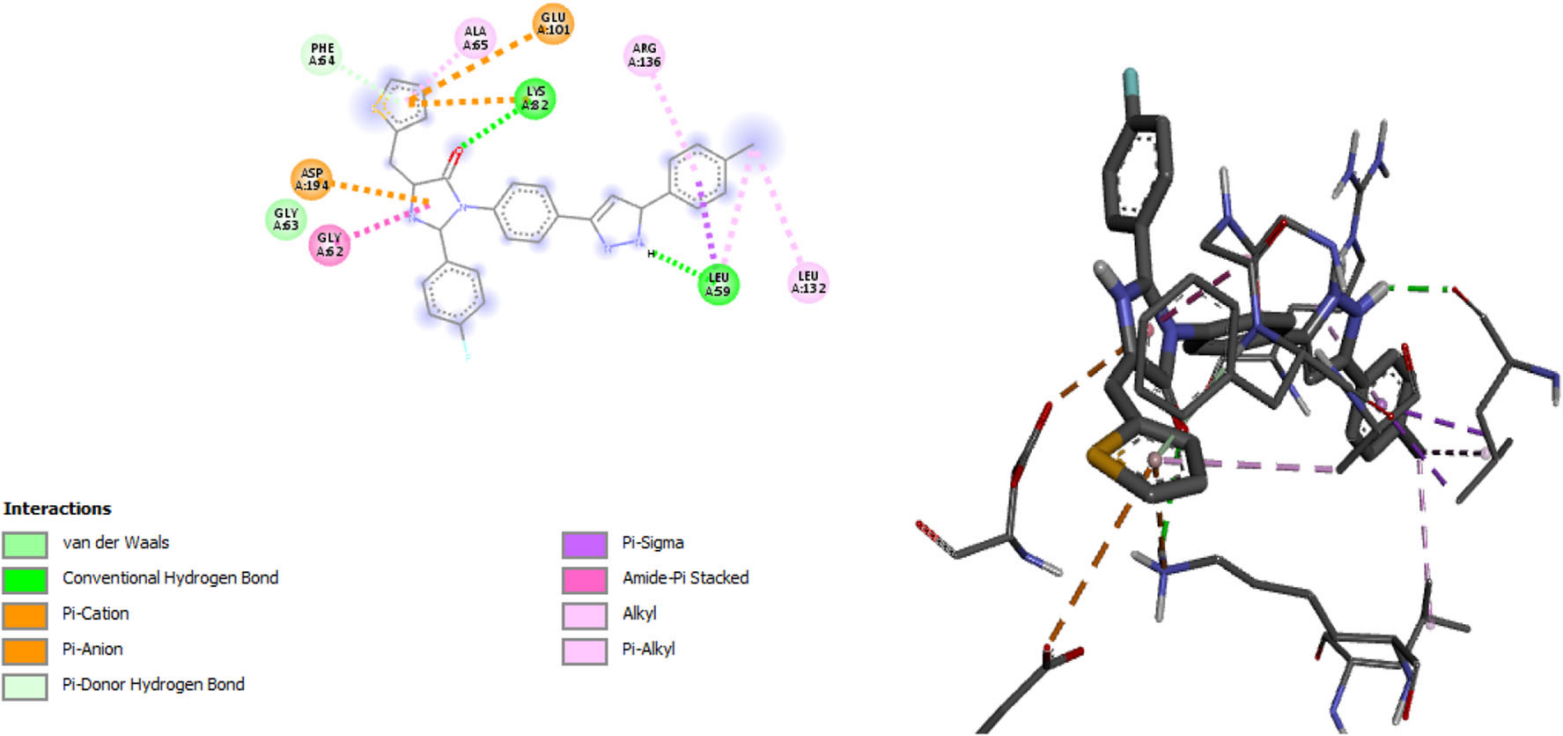
2D and 3D representation of complex 27

**Fig. 6:**
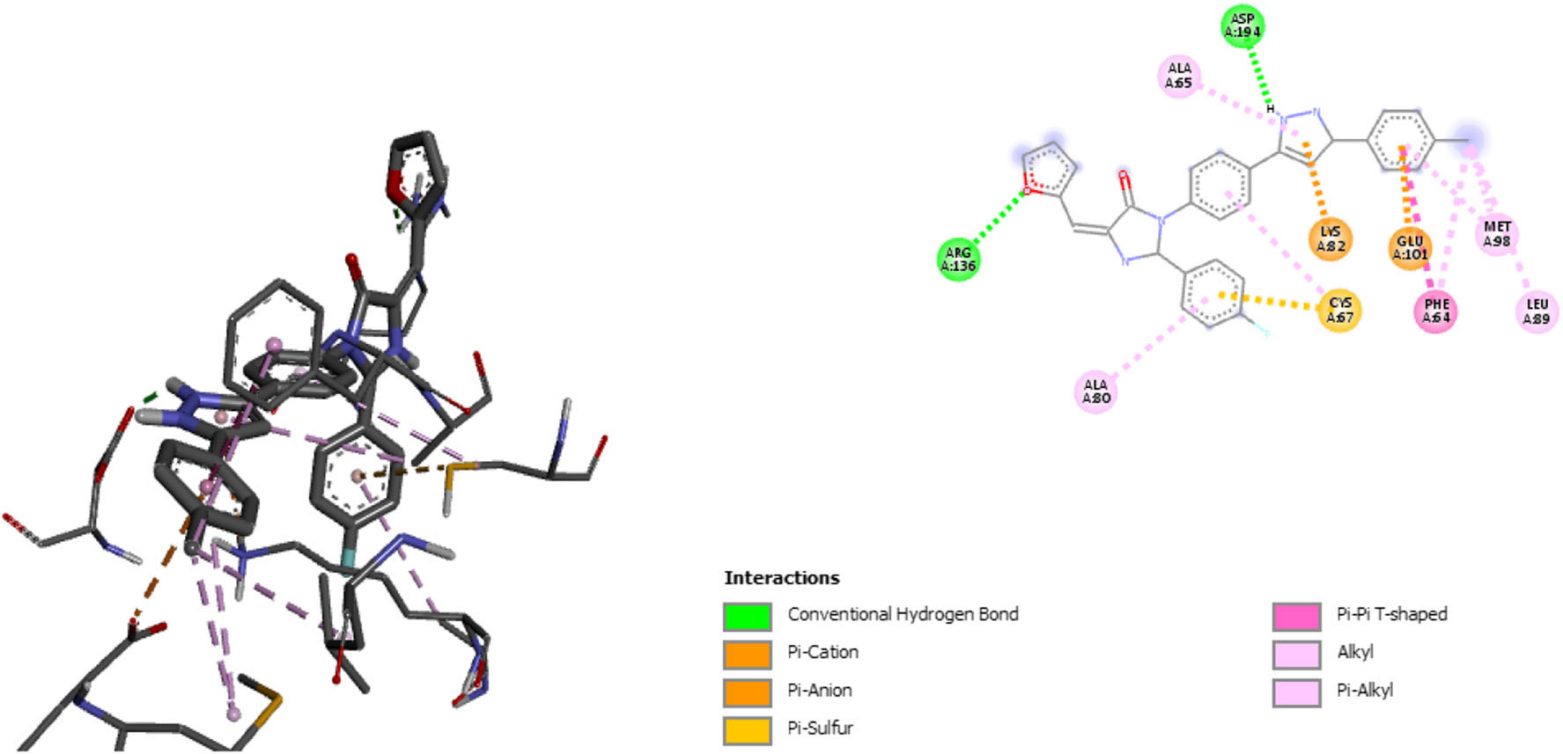
3D and 2D representation of complex 24

A summary of the relationship between some 2-(4-fluorophenyl) imidazole-5-ones derivatives and the receptor was shown in Table 8. The pictorial analysis of docked compounds was accessed by assessing the hydrogen bond interactions, hydrogen bond length, and hydrophobic interactions. Both 2D representations of the binding pose of compounds 24 and 27 to the active pocket of the protein target are shown in Figs. 5 and 6 respectively.

**Table 8:** molecular docking interaction in some complexes

Complex	Binding affinity (kcal/mol)	Amino acid	Bond type	Interaction	Distance(A^0^)
27	−9.1	LYS82	Hydrogen Bond	Hydrogen Bond Interaction	2.76853
		LEU59	Hydrogen Bond	Hydrogen Bond Interaction	2.08894
		LYS82	Electrostatic	Pi-Cation	4.57869
		GLU101	Electrostatic	Pi-Anion	4.83015
		ASP194	Electrostatic	Pi-Anion	3.54145
		PHE64	Hydrogen Bond	Pi-Donor Hydrogen Bond	2.84892
		LEU59	Hydrophobic	Pi-Sigma	3.8345
		LEU59	Hydrophobic	Pi-Sigma	3.93488
		GLY62	Hydrophobic	Amide-Pi Stacked	3.80969
		LEU59	Hydrophobic	Alkyl	4.89515
		LEU132	Hydrophobic	Alkyl	4.61692
		ARG136	Hydrophobic	Pi-Alkyl	4.58314
		ALA65	Hydrophobic	Pi-Alkyl	4.75156
24	−8.8	ARG136	Hydrogen Bond	Conventional Hydrogen Bond	2.60978
		ASP194	Hydrogen Bond	Conventional Hydrogen Bond	1.99045
		LYS82	Electrostatic	Pi-Cation	3.69094
		GLU101	Electrostatic	Pi-Anion	4.16479
		CYS67	Others	Pi-Sulfur	4.37876
		PHE64	Hydrophobic	Pi-Pi T-shaped	5.25545
		LEU89	Hydrophobic	Alkyl	4.82227
		MET98	Hydrophobic	Alkyl	4.1282
		PHE64	Hydrophobic	Pi-Alkyl	4.85386
		CYS67	Hydrophobic	Pi-Alkyl	5.47383
		ALA65	Hydrophobic	Pi-Alkyl	5.17103
		MET98	Hydrophobic	Pi-Alkyl	5.26908
		ALA80	Hydrophobic	Pi-Alkyl	5.10978

### Ligand Base drug design

Ligand based approach was used in designing 18 new imidazole derivative compounds with higher calculated activities than that of the template compounds as shown in Table 9.

**Table 9:**
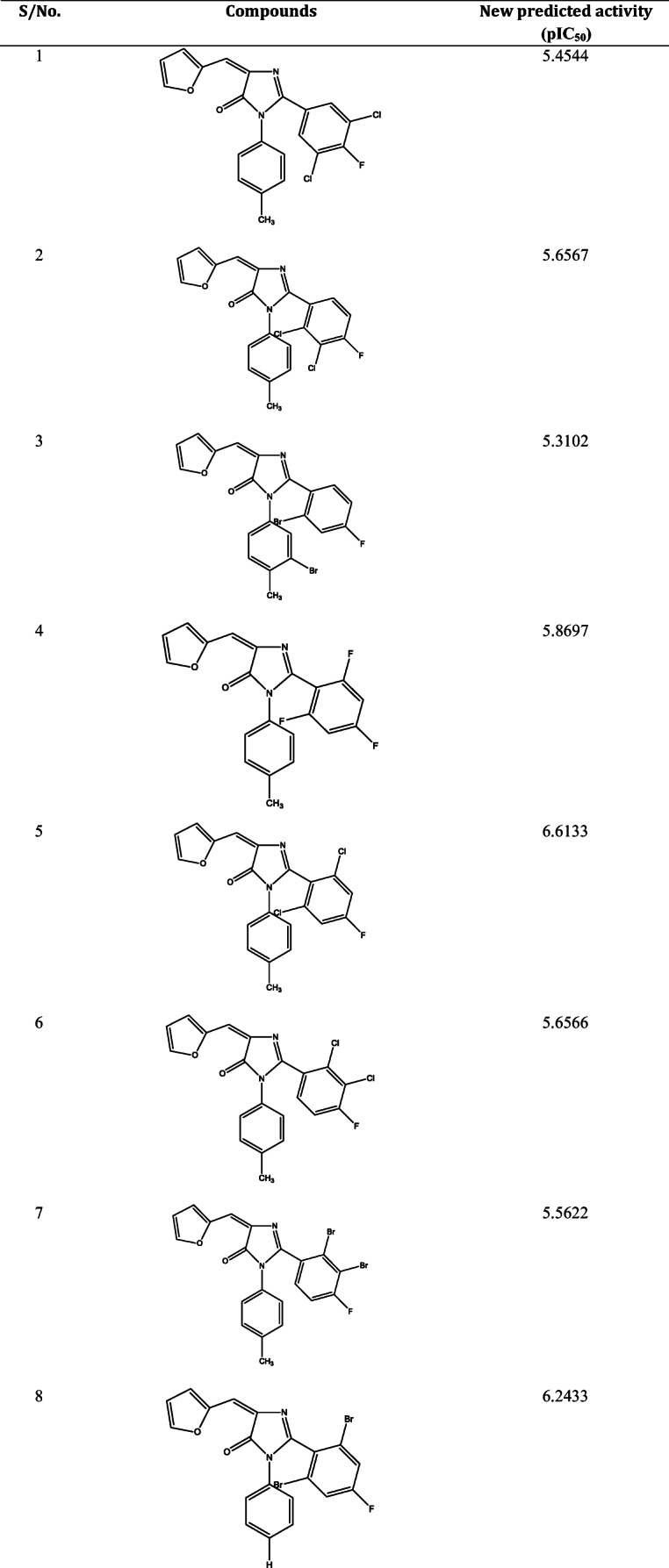

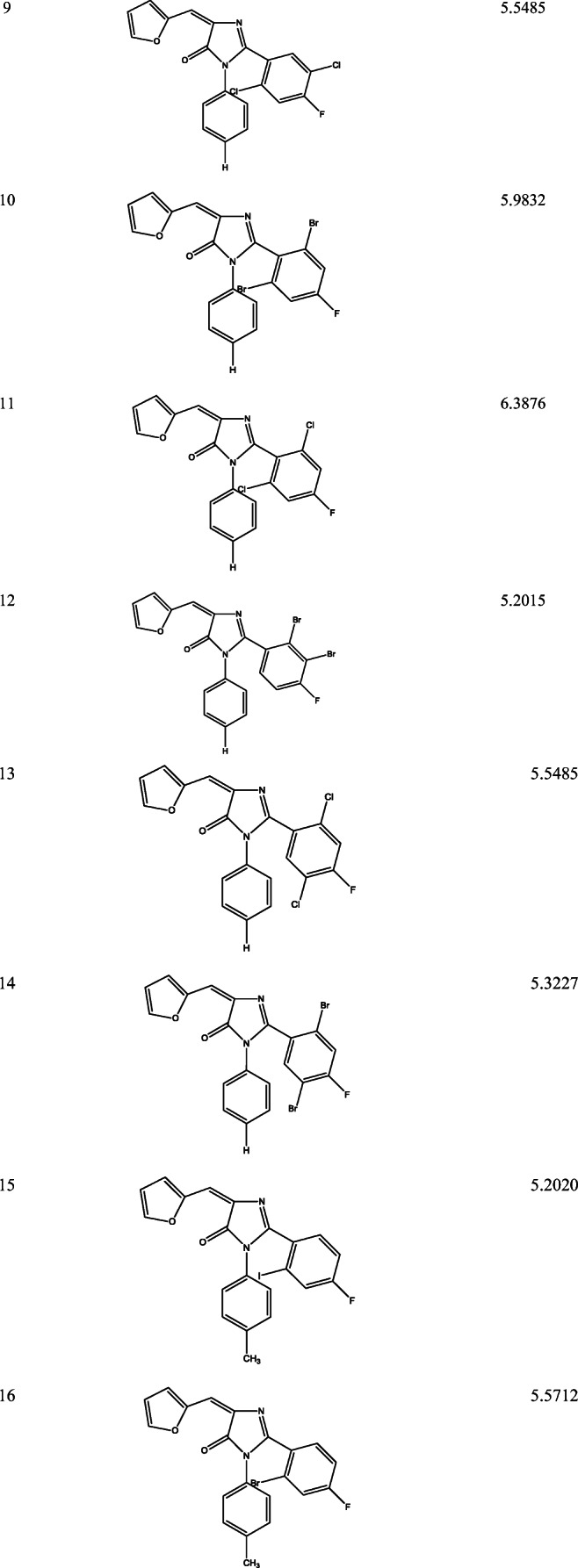

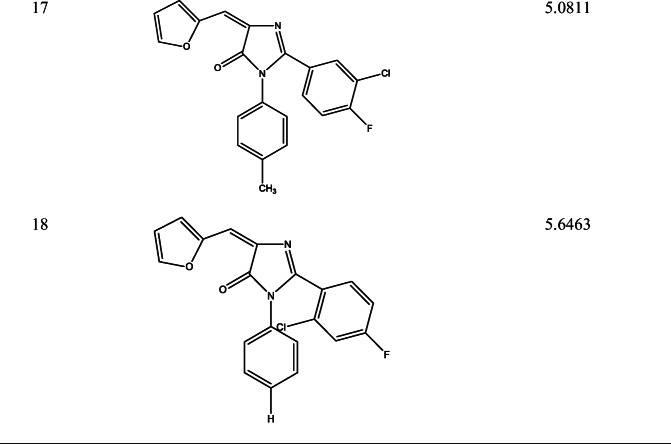
Newly designed imidazole derivative compounds with their new predicted activities (pIC_50_)

### Pharmacokinetics of designed 2-(4-fluorophenyl) imidazole-5-ones compounds

**Fig. 7:**
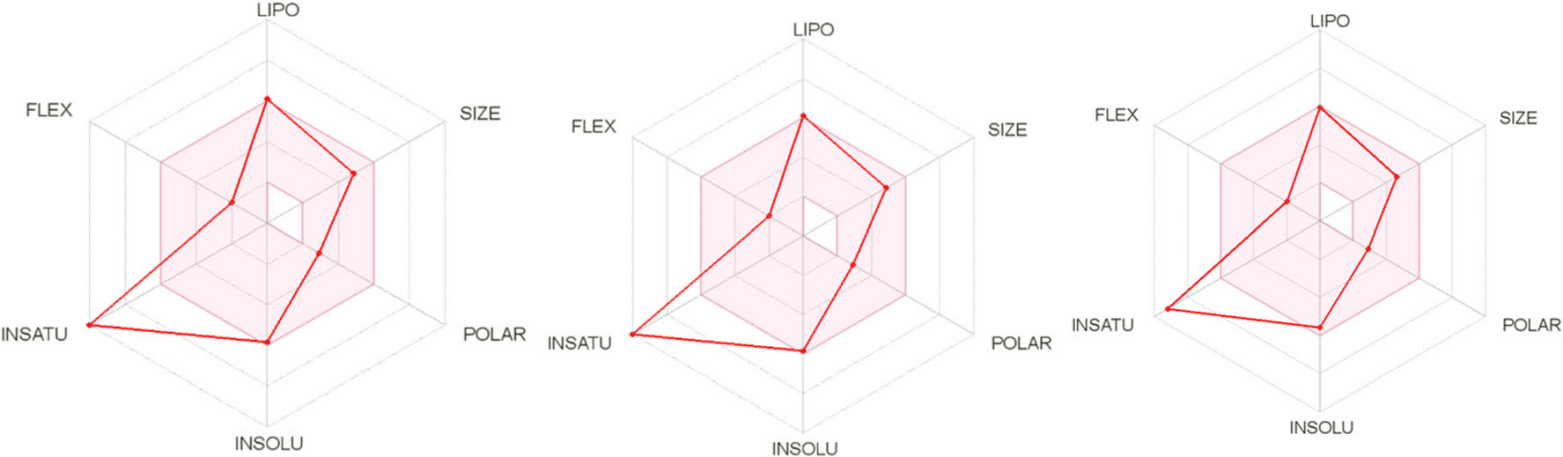
The bioavailability radar for molecules 11, 13, and 17

The newly designed compounds were further explored to ascertain their drug-friendliness. The pharmacokinetic analysis of the new compounds are shown in Table 10, all the compounds pass the Lipinski rule of five test. The bioavailability radar of molecules 11, 13, and 17 are shown in Fig. 7.

**Table 10:** Pharmacokinetics of designed 2-(4-fluorophenyl) imidazole-5-ones compounds

S/No.	MW (mg/mol)	nAH	nRB	HBA	HBD	MR	TPSA (Å^2^)	iLOGP	BBB	PAIN	Brenk
1	415.24	17	3	4	0	114.8	45.81	3.81	Yes	1	2
2	415.24	17	3	4	0	114.8	3.72	3.72	Yes	1	2
3	504.15	17	3	4	0	120.18	45.81	3.93	Yes	1	1
4	382.34	17	3	6	0	104.69	45.81	3.52	Yes	1	1
5	415.24	17	3	4	0	114.80	45.81	3.70	Yes	1	1
6	415.24	17	3	4	0	114.80	45.81	3.72	Yes	1	2
7	504.15	17	3	4	0	120.18	45.81	3.80	Yes	1	2
8	490.12	17	3	4	0	115.21	45.81	3.51	Yes	1	1
9	401.22	17	3	4	0	109.83	45.81	3.49	Yes	1	2
10	490.12	17	3	4	0	115.21	45.81	3.51	Yes	1	1
11	490.12	17	3	4	0	115.21	45.81	3.74	Yes	1	2
12	401.22	17	3	4	0	109.83	45.81	3.35	Yes	1	1
13	490.12	17	3	4	0	115.21	45.81	3.70	Yes	1	2
14	401.22	17	3	4	0	109.83	45.81	3.49	Yes	1	2
15	472.25	17	3	4	0	117.49	45.81	3.67	Yes	1	2
16	425.25	17	3	4	0	112.48	45.81	3.76	Yes	1	1
17	380.80	17	3	4	0	109.79	45.81	3.72	Yes	1	1
18	366.77	17	3	4	0	104.82	45.81	3.29	Yes	1	1

*MW* Molecular weight (<500 mg/mol), *nAH* number of aromatic heavy atoms, *nRB* rotatable bonds, *HBA* Hydrogen bond acceptors, *HBD* Hydrogen bond donors, *MR* molecular refractivity, *TPSA* Topological polar surface area, *BBB* blood-brain barrier

## Discussion

### QSAR of 2-(4-fluorophenyl) imidazol-5-ones derivatives

The results obtained from the QSAR analysis showed that both internal and external validation of the model were in agreement with the minimum proposed values used in assessing the equation as seen in Table 2 above. The external validation of model 1 was achieved using the model parameters from the validation compounds as seen in Tables 3 and 4. The effectiveness of the equation was measured by the reliability of the validation compounds and calculated pIC_50_ of the calibration compounds. The biological, calculated and the residual values of 2-(4-fluorophenyl) imidazol-5-ones compounds are seen in Table 5. The low residual values are obtained from the difference between the biological and calculated activities, showing the high prediction power of eq. 1. Both internal and external validation conforms eq. 1 to be greatly effective, strong, and extremely predictive. Table 6 shows the definition of the model 1 parameters. The mean effect obtained from the model parameters, shows (GATS5e, MATS4e, and SpMax4_Bhs) carries a positive coefficient showing that an increase in those factors would increase the bioactivities of the derivatives while (RDF150u) carrying a negative coefficient indicates a decrease in the descriptor would also increase the experimental activities of 2-(4-fluorophenyl) imidazol-5-ones derivative compounds. The statistical analysis shows that there is no much collinearity amongst the model parameters ensuring that the equation is highly robust as seen in Table 7.

The graph of calculated activities (pIC_50_) against biological activities (IC_50_) as shown in Fig. 1 indicates the pIC_50_ has been in good agreement with the biological activities as seen in Table 3. Figure 2 shows the values of both calibration and validation compounds spread on both sides of the graph, showing no systematic errors between the standardized residual versus bio-activities (Experimental activity) (Jalali-Heravi and Kyani 2004). Fig. 3 shows William’s graph (standardized residuals against leverages), indicating that all the molecules fell in the warning leverage area, calculated to be (h = 0.56).

### Molecular docking analysis

The docking analysis on compounds of 2-(4-fluorophenyl) imidazole-5-ones derivatives with the protein target, Polo-like kinase 1(PKL1) in complex with B16727 was performed. 5 compounds with high pIC_50_ were chosen for these studies, amongst the 5, compound 24 and 27 had the highest docking score of −8.8 and − 9.1 kcal/mol as seen in Table 8.

Compound 27 showed backbone conventional hydrogen bonding interaction between -NH group with LYS82 (2.7685A^0^) and carbonyl group with LEU59 (2.0889A^0^). Three amino acids showed electrostatic interaction at LYS82 (4.57869A^0^) which is a pi-orbital cation interaction then GLU101 (4.83015A^0^), ASP194 (3.54145A^0^) are pi-orbital anion interaction. Furthermore, the compound formed a hydrophobic bond with three amino acids of LEU59 at distance 3.8345A^0^, 3.93488A^0^, and 4.89515A^0^, then GLY62 (3.80969A^0^), ARG136 (4.58314A^0^), and ALA65 (4.75156A^0^).

Again in compound 24, ARG136 and ASP 139 gave covalent hydrogen interaction at a distance of 2.60978A^0^ and 1.99045A^0^. Two electrostatic bonds occur with the compound at LYS82 (4.16479A^0^) and ASP194 (1.99045A^0^). It formed a hydrophobic bond with PHE64 (5.25545A^0^), MET98 (4.1282A^0^) also, the pi-orbital containing delocalized electrons in the benzene ring interact with the alkyl groups of PHE64 (4.85386A^0^), CYS67 (5.47383A^0^), ALA65 (5.17103A^0^), MET98 (5.26908A^0^), ALA80 (5.26908A^0^) three amino acids of PRO384 (5.1107A^0^, 4.7845A^0^ and 4.7531A^0^) to form a hydrophobic bond. Both the hydrogen bond and the hydrophobic interactions in the complexes showed that ligand 24 and 27 of 2-(4-fluorophenyl) imidazole-5-ones derivatives are most active against Polo-like kinase 1(PKL1) in complex with B16727 respectively.

### Ligand based design

Eighteen (18) new 2-(4-fluorophenyl) imidazole-5-ones derivative compounds were designed, their predicted activities were higher than that of the chosen template (compounds 4 with pIC_50−_5.0620 and compound 2 with pIC_50_–4.9846) as shown in Table 9. From the mean effect of the descriptors, SpMax4_Bhs had a greater positive impact followed by GATS5e and MATS4e while RDF150u had the least negative impact on the model. According to MATS4e (Moran autocorrelation - lag 4 / weighted by Sanderson electronegativities) and GATS5e (Geary autocorrelation - lag 5 / weighted by Sanderson electronegativities) descriptors, adding more electronegative elements (GATS5e and MATS4e) would increase the potency of the designed compounds. The modification occurred by adding more electronegative elements to the template (compounds 2 and 4).

### Pharmacokinetics of designed 2-(4-fluorophenyl) imidazole-5-ones compounds

The designed compounds were assessed for their drug-friendliness. The molecules passed the drug- friendliness assessment (ADME and physicochemical properties) as shown in Table 10, none of the designed compounds violated two rules out of the Lipinski rule of five; a famous benchmark utilized in invalidating the drug-likeness of a molecule (as stated in “Molecular Docking Studies” section). The bioavailability radar of molecules 11, 13, and 17 are shown in Fig. 7. The bioavailability radar gives a quick and easy summary of the pharmacokinetic properties of a compound. The pink area signifies the ideal ranges for each property (lipophilicity: XLOGP3 from −0.7 to +5.0, size: molecular weight from 150 to 500 g/mol, polarity: TPSA from 20 to 130 Å^2^, solubility: log S less than 6, saturation: the fraction of carbons in the sp^3^ hybridization not higher than 0.25, and flexibility: less than 9 rotatable bonds) (Daina et al. 2017).

## Conclusion

2-(4-fluorophenyl) imidazole-5-ones derivatives showed a more reliable anti-cancer drug candidate against MCF-7 cell line using QSAR analysis, molecular docking assessment, and pharmacokinetics analysis. The model 1 parameters obtained from QSAR showed that increasing MATS4e and RDF150u, and decreasing GATS5e and SPMAX4_Bhs would proliferation the biological activities of the inhibitors 2-(4-fluorophenyl) imidazol-5-ones derivatives as an effective drug for curing breast cancer. The strength and predictive capability of the generated equation was explored for both internal and external validation assessment which conforms with the least approved values, indicating that model number one parameters could be used in developing new 2-(4-fluorophenyl) imidazol-5-ones drug compounds with higher effectiveness. The model parameters (MATS4e and GATS5e) had more significant and based on their mean effect, adjustment were made on the fragments of the lead compounds (2 and 4) to design 18 new imidazole derivative compounds with a higher calculated activity against MCF-7 cell line.

The molecular docking result showed that compound 24 and 27 had the highest docking score of −8.8 and − 9.1 kcal/mol. From the research it is proved that some series of 2-(4-fluorophenyl) imidazol-5-ones derivatives compounds bind tightly to the binding pose of the target, stabilizing the receptor Polo-like kinase 1(PKL1) in complex with B16727 which is proven from the complexes as seen above. The compounds would serve as the most capable inhibitors against (PKL1) and this shows a revolution in medicine to design and develop new estrogen-positive (MCF-7 cell line) breast cancer drugs.

Additionally, the pharmacokinetics analysis (drug-likeliness test) executed on the designed molecules revealed that all the compounds can move on to the next step of pre-clinical trial because they passed drug-friendliness analysis (ADME and other physicochemical properties) and they also adhered to the Rule of Five: a benchmark used in assessing the drug-likeness of compounds. This shows a great discovery for medicine in finding permanent solutions to breast cancer (MCF-7 cell line).

## Data Availability

The data set was obtained from (Abo-Elanwar et al. 2019).
